# Multimodal Fusion of Chest X-Rays and Blood Biomarkers for Automated Silicosis Staging

**DOI:** 10.3390/jcm14228074

**Published:** 2025-11-14

**Authors:** Blanca Priego-Torres, Iris Sopo-Lambea, Ebrahim Khalili, Ana Martín-Carrillo, Antonio Campos-Caro, Antonio León-Jiménez, Daniel Sanchez-Morillo

**Affiliations:** 1 Department of Automation Engineering, Electronics and Computer Architecture and Networks, School of Engineering, University of Cadiz, 11519 Cadiz, Spain; iris.sopo@uca.es (I.S.-L.); ebrahim.khalili@uca.es (E.K.); ana.martin@uca.es (A.M.-C.); 2 Biomedical Research and Innovation Institute of Cadiz (INiBICA), 11009 Cadiz, Spain; antonio.campos@uca.es (A.C.-C.); antonio.leon.sspa@juntadeandalucia.es (A.L.-J.); 3 Genetics Area, Biomedicine, Biotechnology and Public Health Department, School of Marine and Environmental Sciences, University of Cadiz, 11519 Cadiz, Spain; 4 Pulmonology Department, Puerta del Mar University Hospital, 11009 Cadiz, Spain

**Keywords:** engineered stone, silicosis, blood biomarkers, chest X-rays, machine learning, deep learning, progressive massive fibrosis

## Abstract

**Background/Objectives**: Silicosis, a fibrotic lung disease, is re-emerging globally, driven by an aggressive form linked to engineered stone processing that rapidly progresses to progressive massive fibrosis (PMF). The standard diagnostic approach, chest X-ray (CXR), is subject to considerable inter-observer variability, making the distinction between simple silicosis (SS) and PMF particularly challenging. The purpose of this study was to develop and validate an automated multimodal framework for silicosis staging by integrating artificial intelligence (AI), CXR images, and routine blood biomarkers. **Methods**: We developed three fusion architectures, early, late, and hybrid, connecting blood biomarker analysis with CXR analysis. Deep learning and conventional (shallow) machine learning models were combined. The models were trained and validated on a cohort of 94 patients with engineered stone silicosis, providing 341 paired CXR and biomarker samples. A patient-aware 5-fold cross-validation was used to ensure the model’s generalizability and prevent patient data leakage between folds. **Results**: The hybrid and late fusion models achieved the best performance for disease staging, yielding an area under the receiver operating characteristic (ROC) curve (AUC) of 0.85. This multimodal approach outperformed both the unimodal CXR-based model (AUC = 0.83) and the biomarker-based model (AUC = 0.70). **Conclusions**: This study reveals that AI-based techniques that utilize a multimodal fusion approach have the potential to outperform single-modality methods have the potential to serve as an objective decision support tool for clinicians, leading to more consistent staging and improved patient management.

## 1. Introduction

Silicosis is defined as a chronic and debilitating pneumoconiosis, etiologically classified within the broad spectrum of Interstitial Lung Diseases (ILDs) [1]. Its pathogenesis is intrinsically linked to prolonged inhalation of respirable crystalline silica, leading to progressive and irreversible pulmonary fibrosis, respiratory failure, and death [2]. Classically associated with high-risk settings like mining and quarrying, silicosis has long been a significant public health challenge. Nevertheless, in recent decades, silicosis has experienced an alarming global resurgence, marking a transition in the primary sources of exposure [3]. This flare-up is directly linked to the growing popularity and industrialized use of engineered stone (ES) surfaces [4]. Given that these compounds, commonly used on kitchen and bathroom countertops, possess an exceptionally high silica content, frequently exceeding 85%, the processing and manipulation of these materials represent a new and significant occupational hazard [5].

The initial pulmonary manifestation, simple silicosis (SS), is characterized by the dissemination of small discrete fibrotic nodules throughout the parenchymal tissue. In addition, the specific etiology associated with ES exposure has been demonstrated to induce a pathologically aggressive disease phenotype, frequently exhibiting a lower latency period and rapid clinical evolution [6]. This path can culminate in the progression to progressive massive fibrosis (PMF), defined by the irreversible coalescence of fibrotic lesions into large conglomerate masses, thus inducing a severe and irreversible impairment of lung function. This progression has been empirically documented to occur in up to 38% of affected individuals, even after permanent cessation of exposure to silica [7].

Given the lack of effective therapy and its irreversible course, prophylactic measures and robust staging protocols are the cornerstone of patient management [8]. Consequently, early identification of the onset of the disease and, critically, its progression to PMF is vital [6]. Unfortunately, early-stage silicosis is often subclinical and remains a significant challenge, as current techniques have notable limitations [9]. The diagnosis of silicosis is based on chest radiographs (CXRs), high-resolution computed tomography (HRCT), and occupational and clinical history [2]. Traditionally, the diagnosis of silicosis has relied mainly on correlating the patient’s occupational exposure history with chest radiographs scored by the International Labour Organization (ILO) system [10]. Although HRCT can detect earlier and subtler parenchymal alterations, its routine use is limited by cost and cumulative radiation exposure [3]. Radiographic interpretation requires an experienced radiologist to identify subtle patterns. Opacities can initially be very small (even 1.5 mm), which can be challenging for the reader [11], making the task subjective and with low sensitivity, especially in the early stages, where the diagnostic performance can be limited by poor-quality chest radiographs and by considerable variability between readers [3,9,12].

Research is increasingly focusing on the identification of biomarkers to support clinical decision-making and cost-effective management of silicosis [13,14,15]. Recent research on peripheral blood biomarkers has demonstrated that systemic inflammatory and cell-turnover markers are present in patients with ES silicosis even years after stopping exposure to silica dust. Ratios such as neutrophil-to-lymphocyte (NLR) and platelet-to-lymphocyte (PLR), along with enzyme levels and composite indices, such as the systemic immune-inflammation index (SII) and the systemic inflammation response index (SIRI), have been shown to increase as silicosis progresses [14]. In addition, specific cytokine levels that can vary over time and be associated with disease diagnosis and prognosis have been identified [16].

Meanwhile, artificial intelligence (AI) has achieved remarkable success in medical imaging tasks [17,18]. More specifically, deep learning methods like convolutional neural networks (CNNs) have been widely applied to the detection of pneumoconiosis [19,20,21,22,23,24,25,26]. However, the related task of disease staging, often marked by its ambiguity [19], remains comparatively less explored [11,21,22,26,27].

While unimodal models using imaging or laboratory data alone have shown strong diagnostic performance [15,19,28], they are fundamentally incomplete. Biological phenomena, such as molecular and cellular alterations, often precede radiological evidence by a significant margin, sometimes by years. Biological markers, such as those found in blood, can provide highly sensitive data on molecular and cellular dysregulation, acting as an early warning system long before structural changes occur. Molecular and cellular alterations, such as inflammatory processes or early fibrotic changes, can be active long before they induce structural changes of a magnitude detectable by conventional imaging like X-rays or CT scans. In addition, imaging provides the indispensable anatomical context for structural impact. Given that modality-level fusion techniques have shown promise in improving predictive accuracy for disease diagnosis and prognosis [29,30], our study is based on this synergy, as neither modality alone can simultaneously capture the structural pathology on chest radiographs and the biochemical changes reflected in blood assays. This multimodal fusion concept has already proven valuable in other respiratory diseases, such as for COVID-19 [31,32] and lung cancer [32]. Despite this success, pipelines that jointly apply radiographic and biochemical data to the staging of silicosis are scarce, representing a critical research gap.

To further understand this area, this study presents a pipeline that combines chest radiography and blood biomarkers. It first extracts high-level feature maps from CXR using a pretrained convolutional neural network-based backbone and then reduces them via partial least Squares discriminant analysis (PLS-DA). Three fusion strategies were compared: early fusion, which concatenates PLS-DA output with normalized biomarker vectors before classification; late fusion, which trains separate classifiers on each modality and averages their probabilities; and hybrid fusion, which combines early fusion scores with late fusion probabilities. Each strategy used shallow machine learning classifiers (support vector machine (SVM), random forest (RF), and category boosting (CatBoost), tuned through nested, group-aware cross-validation to ensure no patient overlap between folds. In a cohort of matched imaging and laboratory records, these multimodal models successfully integrated complementary structural and biochemical information to outperform unimodal baselines.

This work, to our knowledge, is the first multimodal approach to specifically differentiate SS from PMF. As such, it provides a novel tool to support the critical staging process and has the potential to enable earlier intervention in occupational respiratory care.

## 2. Materials and Methods

### 2.1. Participants

A total of 94 male workers participated in this study. Inclusion criteria included: (a) a documented occupational history of polishing, cutting, or finishing engineered stone; (b) a diagnosis of silicosis; (c) the availability of time-aligned pairs of chest X-ray images and blood test data; and (d) a signed informed consent. All participants were diagnosed and followed up in the Puerta del Mar University Hospitals in Cádiz, Spain. Clinical, demographic data, and chest radiographs were gathered from clinical hospital records, retrospectively from 2009 to 2016, and prospectively from 2017 to 2024.

### 2.2. Image and Clinical Data

Longitudinal follow-up data were compiled into a single dataset for a unified analysis. The diagnosis was established based on a combination of: (a) a documented history of occupational silica exposure; (b) characteristic findings on chest radiograph according to the ILO classification; and (c) histopathological confirmation via lung or mediastinal lymph-node biopsy in selected cases. In PMF cases where large opacities were not evident on CXR images, HRCT confirmed the diagnosis and stage according to the international classification of high-resolution computed tomography for occupational and environmental respiratory diseases (ICOERD) [33].

### 2.3. Demographics and Respiratory Data

The demographic and clinical information of the participants was obtained from their medical records or through in-person interviews conducted during medical consultations. The data collected encompassed, among other variables, the age at initial exposure, the age at first diagnosis, and the time exposed to ES, measured in years.

Pulmonary function tests were conducted by experienced personnel with standardized spirometry and diffusion (DLCO) equipment, including MasterScreen PFT/Body System (Jaeger-Viasys, CareFusion, Höchberg, Germany) and EasyOne Pro system (ndd Medizintechnik AG, Zürich, Switzerland). Data gathered comprised the forced expiratory volume over 1 s (FEV_1_), forced vital capacity (FVC), FEV_1_/FVC ratio, and lung diffusing capacity for carbon monoxide (DLCO), as determined by the single-breath method in line with global guidelines.

### 2.4. Biochemical and Hematological Blood Markers

Twenty-one biochemical markers were derived from overnight-fasting blood samples. These specimens were placed into ethylenediaminetetraacetic acid (EDTA) tubes and promptly processed for comprehensive hematological and biochemical evaluation by the Clinical Analysis Department at the Puerta del Mar University Hospital.

The total white blood cell (leukocytes) and platelet absolute count were measured, as well as the absolute counts and percentages of neutrophils, eosinophils, basophils, monocytes, and lymphocytes. The white blood cell (WBC) ratios were calculated, including the PLR, the NLR, the lymphocyte-to-monocyte ratio (LMR), the SII (neutrophil × platelet/lymphocyte ratio), the SIRI (neutrophil × monocyte/lymphocyte ratio), and the aggregate index of systemic inflammation (AISI) (neutrophil × monocyte × platelet/lymphocyte ratio). Enzymatic biochemical parameters, including alkaline phosphatase (ALP), lactate dehydrogenase (LDH), and angiotensin-converting enzyme (ACE), were excluded from analysis due to a high percentage of missing values (20% or greater). This left 18 biomarkers, which were used alongside the paired chest X-ray images to train and validate the models.

### 2.5. Dataset

The dataset included a total of 341 chest X-ray images, among which 187 images depicted SS cases while 154 were associated with PMF. Each X-ray image was accompanied by the 18 blood biomarker measurements described in the previous subsection.

### 2.6. Ethics

This study was conducted following the Declaration of Helsinki and approved by the Research Ethics Committee of the Province of Cádiz, Spain (register numbers 151.22, 90.18, 157/16-SIL-2016-01, and 06.20). The Sistema Sanitario Público de Andalucía (SSPA) Biobank of the Hospital Universitario Puerta del Mar (Cádiz, Spain) coordinated the collection, processing, and management of samples and clinical data according to the standard procedures established for this purpose. Informed consent was obtained from all subjects involved in the study.

### 2.7. Multimodal Fusion Staging Methods

**Figure 1:**
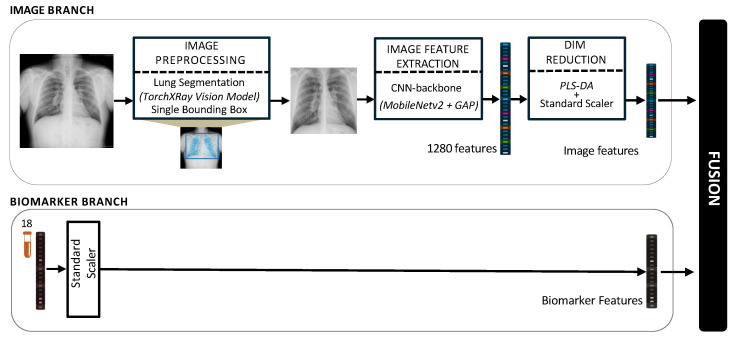
Architecture of the proposed pipeline for the multimodal fusion of chest X-rays and blood biomarkers for the clinical support of silicosis staging.

Modality-level fusion techniques, including the early, intermediate, and late fusion, have demonstrated significant potential to improve predictive precision in various healthcare domains, particularly in the diagnosis and prognosis of diseases [29,30]. In this study, a multimodal framework for staging of silicosis is proposed, as illustrated in Figure 1.

#### 2.7.1. Image Preprocessing

In order to prepare the images for use with the deep learning architecture, a multi-step preprocessing pipeline was developed to standardize all radiographic inputs and concentrate the model analysis solely on the pertinent pulmonary areas. Research has demonstrated that classification based on segmented lung images yields improved results compared to using entire radiographic images [34], and has been used very recently with CXRs [35,36]. An ablation study was performed to assess the impact of the segmentation approach, comparing two distinct techniques, as detailed below.

**Figure 2:**
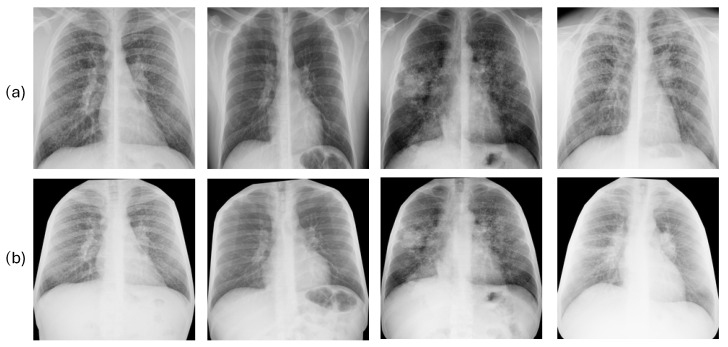
Results of the lung segmentation using the method 1 or standard lung bounding box cut (**a**) and the method 2 or anatomical rib-segmentation (**b**). Each column corresponds to a single patient.

**Method 1**. Standard Lung Bounding Box. The process included two steps. Initially, each original chest radiograph was processed with a pretrained deep learning segmentation model from the TorchXRayVision library [37], specifically trained to generate pixel-level (binary) masks that delineate the exact contours of the lung fields. Using this generated mask, we computed the smallest possible axis-aligned bounding box algorithmically. This involved determining the minimum and maximum y-coordinates and x-coordinates of all pixels classified as lung, resulting in the tightest rectangle enclosing both lung fields. The initial radiograph was subsequently cropped based on these specific coordinates. Focusing on the lungs in this manner can eliminate distracting information and unnecessary anatomical details (such as shoulders, image edges, and annotations) that might result in misleading correlations. In addition, it standardizes the images, mitigating the effects of differing patient positioning, body habitus, and imaging field-of-view across the dataset. After the cropping stage, the resulting images were of variable sizes, depending on the patient’s lung shape and the original bounding box. To create a uniform input for the deep learning model, each cropped image was subsequently resized to a fixed size of 224 × 224 pixels, the standard input size for the deep learning architecture. Finally, each image was converted to 3-channel RGB. The resulting 3-channel image array included 8-bit integer pixel values in the [0, 255] range. It was then passed to the dedicated MobileNetV2 preprocess input function. This final step applied the specific ImageNet-based normalization (e.g., scaling pixel values to [−1, 1]) required by the pretrained network. Figure 2a illustrates the result of applying this segmentation approach to some images in our dataset.

**Method 2**. Anatomical Rib-Segmentation. We implemented an anatomical segmentation approach. Details can be accessed in [28]. This technique uses rib segmentation and keypoint alignment to produce anatomically coherent augmentations, ensuring structural integrity and enhancing the generalization capabilities of the model. This approach guarantees precise transformation estimation and the alignment of thoracic areas while maintaining anatomical consistency. It enables the assessment of the pulmonary region and other tissues, such as the mediastinum. This is significant because adjacent areas such as the heart, pleura, and large blood vessels can also reveal changes, providing information on silicosis-related complications. After segmentation, images were resized to 224 × 224 pixels and converted to 3-channel RGB, as detailed in method 1. Figure 2b displays the outcomes produced by this segmentation technique on several images from our dataset.

**Ablation Study**. To justify our final choice of the lung segmentation approach, an ablation study was conducted. We hypothesized that the anatomical context preserved by the anatomical rib-segmentation method (method 2) contains relevant diagnostic information absent in the standard lung bounding box segmentation (method 1). To test this, we compared the performance of unimodal image-based models (trained only on CXR data) processed with method 1 versus method 2. The method that yielded the best unimodal performance was selected and used for all subsequent multimodal fusion experiments.

#### 2.7.2. Image Feature Extraction and Dimensionality Reduction

For image-based feature extraction, we used the MobileNetV2 architecture [38]. It was selected for its high computational efficiency and proven efficacy as a transfer learning backbone. The backbone was initialized with ImageNet weights. An important methodological decision in our study was to maintain the entire convolutional backbone in a frozen state. This implies that all the pre-learned weights in the MobileNetV2 layers remained unchanged during the training of our model. This strategy was important, given our specialized, moderately sized dataset, as it helps prevent overfitting by using the complex network as a static, robust feature extractor rather than retraining it from the beginning. The network was truncated before its final classification head. To adapt the pretrained network to our silicosis staging task, we removed the original 1000-class fully connected layer and replaced it with a task-specific output layer.

The preprocessed images were input into this fixed backbone and underwent a series of convolutional operations. MobileNetV2 is distinguished by its inverted residual blocks that utilize depthwise separable convolutions, allowing for the gradual encoding of structural and textural patterns at various scales. The deep feature hierarchy reaches its peak with a global average pooling (GAP) layer. This layer consolidates the spatial activations from the last feature map, which is sized at 7 × 7 × 1280, by taking their average. This process results in a fixed-length feature vector of 1280 dimensions. This vector provided a compact, semantic representation of the radiograph, encapsulating its visual details for subsequent fusion and analysis.

Rather than appending additional trainable neural layers to the 1280-dimensional MobileNetV2 features, which can be prone to overfitting with moderately sized datasets, we applied PLS-DA to the feature vectors derived from the GAP layer. PLS-DA is a robust supervised dimensionality reduction technique [39].

To prevent data leakage, this entire preprocessing pipeline was integrated into our patient-aware cross-validation framework. For each fold, all scaling and transformation parameters were fitted exclusively on the training subset using the following three-phase approach:

- Initial Feature Scaling: A standard scaler was fitted to the 1280-dimensional training features. Then, this scaler was used to transform both the training and test sets.
- Latent Space Transformation: A PLS-DA model was fitted on the scaled training features and labels (SS vs. PMF). The optimal number of latent components was determined to be 15 via a separate nested cross-validation, as this provided the best balance between explained variance and model stability. Then, this fitted PLS-DA model was used as a transformer, projecting both the training and test sets into a 15-dimensional latent space.
- Final Latent Scaling: A second standard scaler was fitted, this time only on the resulting 15-dimensional PLS scores from the training set. This final step normalized the new latent features and was then applied to the 15-dimensional test set scores.

This double-standardization process was essential to ensure the final feature representations were stable and uniformly scaled across all cross-validation folds before being passed to the fusion models.

#### 2.7.3. Biomarkers Processing

Concurrently, the 18-dimensional biomarker data was processed independently. To normalize the heterogeneous scales of these features, a standard scaler was fitted. As with image features, this scaler was fitted solely on the training data within each cross-validation fold and then applied to both the train and test sets. This process prevents data leakage and ensures an unbiased performance estimate.

#### 2.7.4. Multimodal Data Integration

Following the preprocessing pipeline, each patient within a given cross-validation fold was represented by two modality-specific feature vectors. The imaging modality was characterized by a vector of 15 scores derived from PLS-DA analysis. The hematological modality was represented by a vector consisting of 18 standardized blood biomarker values. Once both modalities were transformed in this fold-aware manner, three complementary fusion strategies were explored, namely (a) early fusion; (b) late fusion; and (c) hybrid fusion.

**Figure 3:**
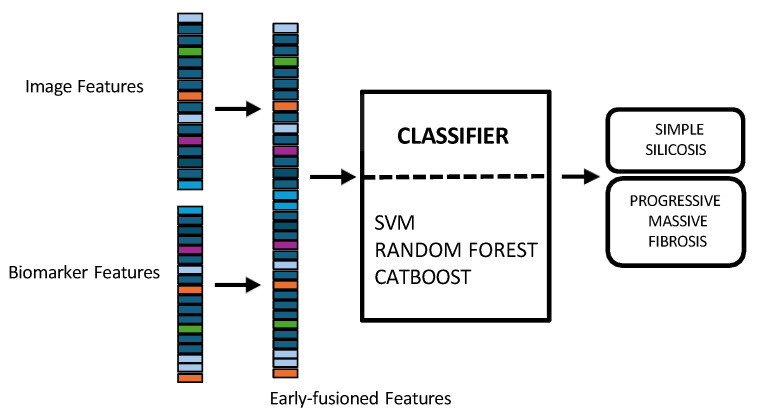
Multimodal early fusion of chest X-rays and blood biomarkers for the clinical support of silicosis staging.

**Figure 4:**
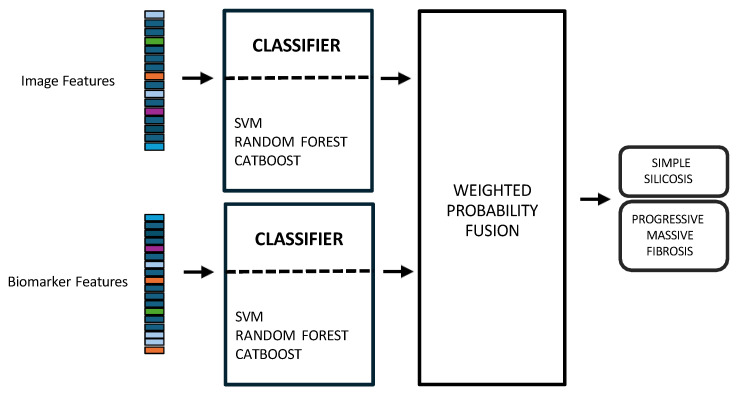
Multimodal late fusion of chest X-rays and blood biomarkers for the clinical support of silicosis staging.

**Figure 5:**
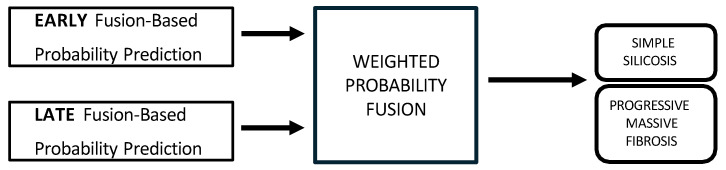
Multimodal hybrid fusion of chest X-rays and blood biomarkers for the clinical support of silicosis staging.

1. In early fusion, features of both modalities are combined before classification, as depicted in Figure 3. This approach has the theoretical advantage of allowing the model to learn cross-modal relationships from low-level features. Nonetheless, this early fusion is prone to overfitting when samples are limited [40] and may struggle to detect connections between the modalities if these connections only become clear at more abstract levels, as marginal representations are not specifically learned [41,42,43]. In this study, the image feature vector, derived from PLS-DA scores, and the biomarker vector were concatenated. This process resulted in a 33-dimensional vector representing each patient at a specific time point. This vector was used as input to train and evaluate three shallow ML models, selected for their different approaches to handling feature spaces: SVM [44], RF [45], and CatBoost [46].
  Model training and hyperparameter optimization were conducted using a nested, group-aware cross-validation framework to ensure robust, patient-level separation.
2. Late fusion combined modality-specific model outputs into a final decision, offering better robustness to missing data, easier interpretability, and simpler integration into clinical workflows. Nevertheless, this strategy may lose some fine cross-modal dependencies and might sacrifice detailed inter-modality relationships [40,42]. In this work, two separate classifiers were trained in parallel: the first model used only the image-based feature vectors, while the second used the standardized biomarker vectors, as illustrated in Figure 4.
  Hyperparameter tuning for both models was performed independently using a nested, group-aware cross-validation procedure. During inference, each modality-specific model generates a probability score for PMF. These two scores were then fused into a single prediction using an adaptive weighting scheme, where the weights were determined by the area under the receiver operating characteristic (ROC) curve (AUC) achieved by each model on the internal validation folds. A final binary classification was made by applying a 0.5 threshold to this weighted-average probability.
3. Hybrid fusion was designed to integrate the output of both the early and late fusion frameworks (Figure 5). Independent classifiers were trained for each modality, and then their probability predictions were combined.

The hybrid approach seeks the best of the early and late strategies, by integrating the outputs of both frameworks. Instead of forcing a single model to learn everything, the hybrid model acts as a meta-learner that learns to weigh the importance of early fusion prediction versus late fusion prediction. This approach operated on two predictive probabilities for each test sample: the output from the early-fusion classifier, and the score from the late-fusion branch. Instead of using a simple average or a complex meta-model, we adopted a dynamic weighting scheme based on performance. We implemented a nested cross-validation procedure that runs within each fold of the main training loop. For each fold, the training set was used to determine the optimal weights for late fusion. The training data of each fold was divided into 3 internal sub-folds. An unimodal image model and an unimodal biomarker model were trained and evaluated separately on these internal divisions. The average performance (AUC) of each branch was calculated across these internal folds. The final weights were assigned in direct proportion to these average AUC:

(1) $$Weight_{Image}=\frac{AUC_{Image}}{AUC_{Image}+AUC_{Biomarker}}$$

(2) $$Weight_{Biomarker}=\frac{AUC_{Biomarker}}{AUC_{Image}+AUC_{Biomarker}}$$

Following, a classification threshold of 0.5 was applied. This method ensured that the most reliable modality in the training data had a greater influence on the final prediction.

To ensure a fair comparison across these three methodologies, the same classifier type (i.e., SVM, RF, and CatBoost) was consistently used for all components within the early, late, and hybrid fusion pipelines. The nested group-aware cross-validation strategy was maintained throughout all experiments to ensure patient-level data separation between training and validation sets.

### 2.8. Unimodal Approaches

**Figure 6:**
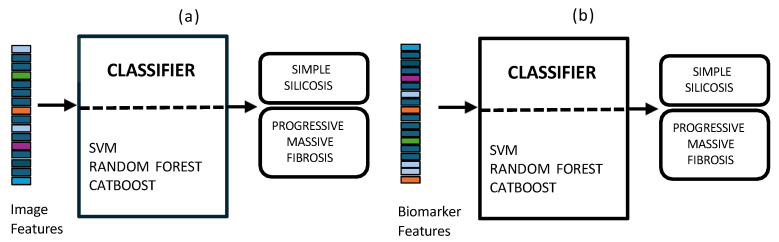
Unimodal approaches using chest X-rays (**a**) and blood biomarkers (**b**) for the clinical support of silicosis diagnosis and staging.

To assess the benefit of data integration, the results of the multimodal approach were compared with those derived from unimodal analyses. The unimodal feature sets, comprising image and biomarker data respectively, were generated following the same pipeline detailed in Figure 1. These separate feature sets were then used to train the shallow ML models (Figure 6). This optimization process was conducted within each training fold to find the optimal hyperparameter combination for each classifier. For the SVM, we enabled probability estimates, a necessary step for our fusion models. The grid search explored combinations of the regularization parameter C [0.1, 1, 10], the kernel gamma coefficient, and the kernel type: radial basis function (RBF) and linear. For the RF ensemble, the search optimized structural parameters, including the number of estimators [100, 200], the max depth of the trees [0, 10, 20], the min samples split [2, 5], and the min samples leaf [1, 2]. Finally, for the CatBoost model, Bernoulli bootstrap type was used, and the subsample rate was fixed to 0.8 to introduce stochasticity. The grid search tuned the number of iterations [100, 200], the learning rate [0.01, 0.1], the tree depth [4, 6, 8], and the regularization term [1, 3, 5].

### 2.9. Performance Metrics and Validation Scheme

A nested 5-fold cross-validation framework was implemented to ensure a robust and unbiased evaluation of all models. To prevent data leakage and ensure that predictions were generalizable across patients, the data folds were created at the patient level. This group-aware strategy ensured that all records from a single patient belonged exclusively to either the training or the testing set within any given fold. The primary splitting strategy employed stratification to maintain the same proportion of SS and PMF cases in each fold. However, if patient grouping constraints made stratification impossible, a standard group K-fold split was used as a fallback. As abovementioned, a secondary inner cross-validation loop was executed within each outer training fold to perform hyperparameter tuning via a group-aware grid search. Once the optimal hyperparameters were identified, the model was retrained on the entire outer training set.

The performance of the retrained model was then assessed on the held-out test fold. For each fold, performance metrics were derived from the four outcomes of the confusion matrix for the PMF class as positive class: True Positives (TP); True Negatives (TN); False Positives (FP); and False Negatives (FN). Based on these components, the following metrics were calculated:

(3) $$\mathrm{Accuracy}=\frac{\mathrm{TP}+\mathrm{TN}}{\mathrm{TP}+\mathrm{TN}+\mathrm{FP}+\mathrm{FN}}$$

(4) $$\mathrm{Precision}=\frac{\mathrm{TP}}{\mathrm{TP}+\mathrm{FP}}$$

(5) $$\mathrm{Recall}=\frac{\mathrm{TP}}{\mathrm{TP}+\mathrm{FN}}$$

(6) $$F1\text{-}\mathrm{score}=2\times \frac{\mathrm{Precision}\times \mathrm{Recall}}{\mathrm{Precision}+\mathrm{Recall}}$$

The AUC was also calculated. To provide a final, aggregate measure of performance, these metrics were averaged across all five folds, and 95% confidence intervals (CI) were estimated. This allowed for a direct and fair comparison between the unimodal baselines and the proposed multimodal fusion strategies.

### 2.10. Statistical Analysis

Post-hoc statistical analysis was applied to determine if the best-performing model was significantly superior to its corresponding unimodal baselines. Wilcoxon signed-rank test was used for this comparison, as it does not assume a normal distribution of the score differences. The test was applied to the paired AUC scores generated by each model on the identical test folds. A *p*-value < 0.05 was considered statistically significant.

Python 3.10 (Python Software Foundation, Wilmington, DE, USA) was used for statistical analysis and for training and validating the models.

## 3. Results

### 3.1. Study Group

The study cohort consisted of 94 patients, all male. The mean age at the time of diagnosis was 37.0 ± 7.5 years. Regarding occupational history, the mean duration of exposure to silica was 13.2 ± 6.1 years. The mean values of the characteristics of lung function of the patients included in the study are detailed in Table 1.

**Figure 7:**
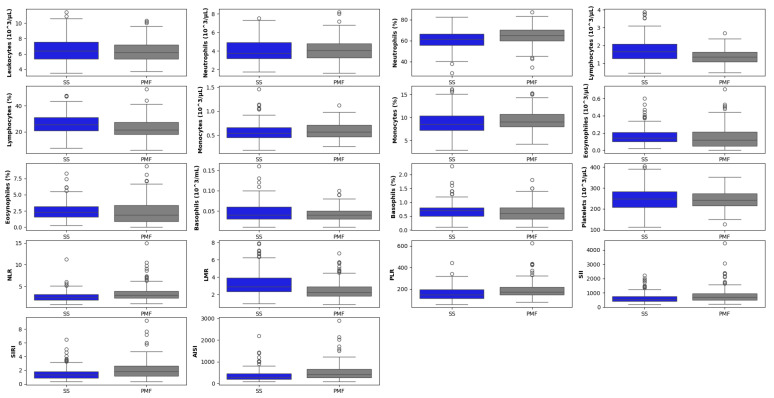
Boxplot matrix of hematological biomarkers segregated by simple groups of patients diagnosed with silicosis (SS) and with progressive massive fibrosis (PMF). The horizontal line within each box represents the median, while the box edges indicate the interquartile range (25th to 75th percentiles). Whiskers extend to the non-outlier range (within 1.5 × IQR). The circles represent outliers, defined as individual data points falling beyond the whiskers.

The distribution of hematological markers, stratified by diagnostic group, is shown in Figure 7.

**Figure 8:**
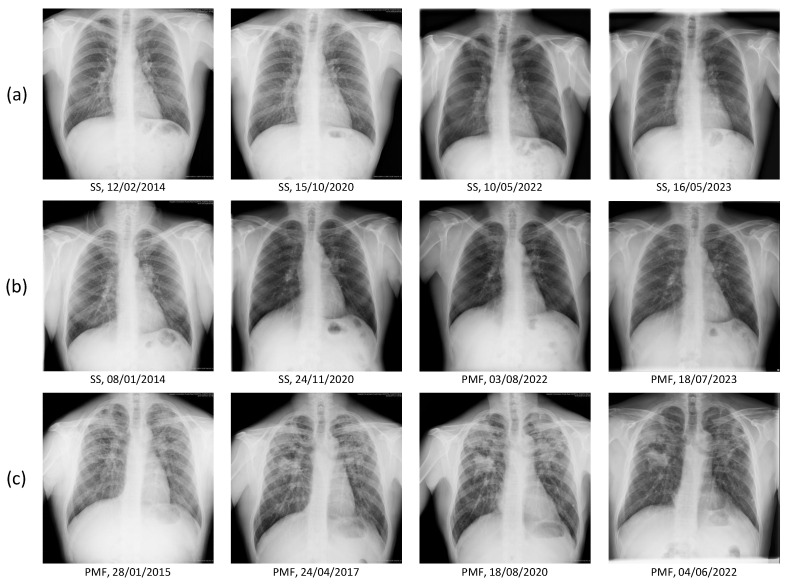
Representative longitudinal chest radiographs from three study participants. Each row corresponds to a single patient. (**a**) Patient initially diagnosed with simple silicosis (SS) and showing stable radiological findings over several follow-up dates. (**b**) Patient with progressive disease course, evolving from SS to progressive massive fibrosis (PMF) during follow-up. (**c**) Patient diagnosed with PMF at baseline and monitored across multiple time points.

Figure 8 presents representative longitudinal chest radiographs: (a) a patient with simple silicosis (SS) showing stable findings over multiple follow-up dates; (b) a patient who progressed from SS to progressive massive fibrosis (PMF) during follow-up; and (c) a patient diagnosed with PMF at baseline and monitored across time.

### 3.2. Ablation Study: Justification of Image Segmentation Method

To quantify the impact of the lung segmentation pipeline, an initial ablation study was conducted. We compared the performance of unimodal, image-only models to determine which segmentation strategy yielded the best results. The baseline method (cropping to the lung bounding box) was compared against the rib-segmentation method, which preserves anatomical context. The results, presented in Table 2, show a clear and significant improvement of the performance when using the latter method (best AUC of 0.84 vs. 0.77).

The best-performing model using this technique (SVM) achieved an AUC of 0.84, a substantial increase from the best-performing standard lung bounding box model (CatBoost/RF), which achieved an AUC of 0.77. This confirms our hypothesis that the anatomical context preserved by method 2 (e.g., mediastinum, pleura) contains critical diagnostic information for staging, which is discarded by the standard lung-only crop. Therefore, based on this evidence, method 2 (anatomical rib-segmentation) was selected for all subsequent multimodal experiments.

### 3.3. Unimodal Models Performance

To establish performance baselines, unimodal models were first evaluated. Table 3 summarizes the performance of the image-based models (using the selected segmentation method) and the biomarker-based models, trained with three different classifiers.

A clear performance gap was observed between the two modalities. The image-based model proved to be the strongest unimodal baseline. The SVM/CatBoost classifiers achieved the best performance in this category, with an AUC of 0.83. In contrast, the biomarker-based model yielded more modest results, confirming it as the weaker of the two modalities. The best classifier for this data was RF, which achieved an AUC of 0.70.

### 3.4. Multimodal Models Performance

Following the evaluation of the unimodal baselines, the fusion strategies were evaluated to determine if the integration of biomarker data could improve upon the strong performance of the image-only model. The results for early, late, and hybrid fusion are presented in Table 4.

The analysis reveals that the choice of the fusion strategy was critical. The early fusion, which relied on simple feature concatenation, did not yield an improvement over the best unimodal baseline. Its top-performing classifier (CatBoost) achieved an AUC of 0.83. In contrast, the more advanced fusion strategies successfully integrated both data sources to achieve superior performance. Both the hybrid fusion and the late fusion models produced the highest discriminative performance of the entire study. These models reached a top mean AUC of 0.85. Notably, the CatBoost classifier was the most effective classifier for both of these top-performing fusion strategies, as well as for the early fusion model. These results demonstrate that a sophisticated fusion of imaging and biomarker data can extract complementary information and provide an advantage over using imaging alone.

To formally validate our findings, a post-hoc analysis was conducted using the Wilcoxon signed-rank test. The analysis confirmed that both advanced fusion strategies were statistically tied as the best-performing models in the study. Both hybrid fusion and late fusion models were found to be statistically superior to the biomarker-only model (*p* < 0.05). Both top models also achieved a higher mean AUC than the strong image-only baseline. However, this performance trend did not reach statistical significance for either the hybrid (*p* = 0.22) or the late model (*p* = 0.40), likely due to the limited statistical power inherent in a 5-fold comparison.

## 4. Discussion

Our study evaluated three different fusion architectures: early (at the feature level), late (at the decision level), and hybrid. Each approach presents an inherent trade-off between information integration and model complexity. The results obtained confirm that integrating structural information from radiographs with systemic information from blood markers provides a more comprehensive feature set, leading to more accurate predictions.

The ablation study confirmed that the surrounding anatomical structures (e.g., mediastinum, pleura) contain relevant diagnostic information for disease staging, which must be preserved in the lung segmentation step. Using this anatomical-rib segmentation, we evaluated the multimodal strategies.

The early fusion approach, which combines features at the input level, was found to be suboptimal. Its best performance (AUC 0.83) did not outperform the image-only unimodal baseline. This suggests that simply concatenating the feature vectors is an ineffective strategy, possibly because the classifier struggles to optimize the disparate feature spaces simultaneously, or because the information from the biomarker vector is drowned out by the image vector. In contrast, the late and hybrid fusion strategies proved to be the most effective. Both approaches achieved the highest performance in the entire study, reaching a top AUC of 0.85 (using the CatBoost classifier). The late fusion scheme provided better accuracy, precision, and F1-score than the hybrid approach. It demonstrates that a gain can be achieved by integrating biomarker data and a more sophisticated fusion strategy. The success of the late fusion strategy highlights the value of modularity. This modularity allows each classifier to be optimized independently. However, its main drawback is that it might miss the opportunity to learn any correlations or interactions between the image and blood modalities before the final decision stage. The hybrid fusion model, which also achieved the top-tier AUC, offered balanced and stable performance across classifiers. This suggests that leveraging the strengths of both feature-level and decision-level integration provides an equally robust pathway to achieving state-of-the-art performance. This analysis confirms that while advanced multimodal fusion (either late or hybrid) provides a significant advantage over using biomarkers alone, the image modality is the dominant driver of performance. The improvement in mean AUC across both top fusion models suggests that multimodal integration is a valuable strategy. A recent systematic review of biomedical multimodal deep learning has shown that intermediate or hybrid fusion approaches, which lie between early and late fusion, often strike the best balance of performance vs. practicality, particularly given real-world issues of missing data, temporal variation, and modality heterogeneity [42]. Our results are aligned to this finding. Despite these benefits, the greater architectural complexity and larger number of parameters demands careful regularization to avoid overfitting.

The development of AI-based models to support the clinical diagnosis of pneumoconiosis has become a highly active field of research in recent years [47]. A substantial portion of this effort has focused on using deep learning architectures, primarily CNN models, applied to CXR for disease screening [11,21,22,25,26,27,28,48,49]. In this task, which typically involves a binary classification, studies have reported excellent performance, demonstrating the ability of these models to identify radiological findings consistent with the disease with high accuracy. However, transitioning from a screening task to one of clinical staging presents considerably greater challenges. The ability of AI models to classify the severity of silicosis has been explored far more sparingly in the literature [11,21,22,26,27]. Studies that have addressed this problem consistently report a decline in model performance compared to screening tasks. This difficulty is often exacerbated in multi-class classification approaches that, by including a category for healthy subjects alongside the different disease stages, can dilute the model’s ability to learn the subtle morphological differences that define silicosis progression.

Furthermore, the diversity in validation approaches and the heterogeneity of the datasets used for model development make a fair and direct comparison between different approaches challenging, complicating any assessment of the true state-of-the-art in disease staging [28]. These gaps underscore the need to develop and validate robust models specifically designed for staging, a challenge that this study directly addresses by considering a multimodal approach, which involves blood biomarkers. To our knowledge, there are no existing multimodal strategies similar to the one introduced here for staging silicosis caused by engineered stone.

Evaluation of common blood biomarkers, such as complete blood counts and particular leukocyte ratios, has been proposed to potentially play an important role in the diagnosis and monitoring of silicosis. These markers can reveal a state of chronic inflammation associated with the disease, making them a valuable and cost-effective support tool for clinicians, potentially helping in early detection, diagnosis, and tracking of the progression of the disease from SS to PMF. Furthermore, inflammatory ratios such as NLR and SII have been reported to increase significantly with the severity of silicosis, providing a more comprehensive view of the balance between the patient’s inflammatory and immune responses [14,15,50]. Therefore, the use of these routine blood biomarkers with AI-based models presents a promising, non-invasive, and accessible approach to enhance the clinical management of silicosis.

Integrating routine blood biomarkers with CXRs using AI, as demonstrated in this study, can enhance the diagnostic framework for silicosis. Blood biomarkers provide information about the systemic inflammatory response and the biological activity of the disease, while CXRs provide vital information about the structure of the lung. By merging these two complementary data sources, AI-driven models can generate a more detailed and comprehensive understanding of a patient’s condition than either method alone. This synergy can lead to improved diagnostic accuracy, facilitate earlier disease detection, and improve differentiation between stages of silicosis, leading to more timely and effective clinical interventions. The results obtained confirm the value of this integrated approach. By learning to identify complex patterns across both the systemic biomarker data and the visual radiographic features, the models based on late and hybrid integration have demonstrated enhanced predictive power and higher diagnostic accuracy compared to models relying on a single data source.

This study has some limitations. The analysis is based on a relatively small sample size from a single hospital center, which may limit the generalizability of the findings. Therefore, validation of models in larger, multicenter cohorts is warranted to confirm their robustness across different clinical settings. The study exhibits a gender bias, as all participants were male. It should be noted, however, that this bias is not a result of selection criteria but rather a reflection of the occupational demographics of the engineered stone industry, an activity traditionally and predominantly performed by men. Our evaluation focused primarily on discriminative performance using a fixed threshold-independent AUC as the main metric. For any future clinical implementation, this threshold would need to be calibrated using a greater cohort to meet specific clinical requirements, such as maximizing sensitivity or specificity for the target population.

Several avenues remain as future works for enhancing diagnostic accuracy. From a methodological perspective, our work establishes a strong baseline using early, late, and hybrid fusion. Meta-learning strategies and more advanced fusion architectures represent a clear next step, as well as evaluating domain-specific models [51,52], both frozen and fine-tuned to further improve the performance of the image branch. For instance, attention-based mechanisms are a feasible technique to allow the model to learn the relative importance of each modality dynamically, potentially improving both performance and interpretability. For future larger-scale studies, multimodal transformers offer a state-of-the-art approach for capturing highly complex, non-linear dependencies between heterogeneous data streams [53,54]. Evaluating these advanced methods, balanced against their data requirements and computational costs, is a promising direction for future research in automated silicosis diagnostics. Additionally, formal clinical utility analysis, such as a decision-curve analysis (DCA) to evaluate the net clinical benefit of models, remains an essential step for future implementation studies. Finally, validating this framework against a silica-exposed, unaffected control cohort, integrating explainable AI techniques, and exploring the within-patient time dynamics by modeling the longitudinal trajectory, are crucial steps to transition the model from a diagnostic staging tool into a real-world triage method.

We addressed a significant gap in multimodal research by systematically comparing early, late, and hybrid fusion architectures. Our findings confirm that for the heterogeneous data used in silicosis staging, late and hybrid fusion strategies are the most effective. This aligns with recent reviews highlighting that these approaches are more practical and reliable [41,42]. The strategy proposed enhances diagnostic accuracy and assists in classifying patients for earlier, personalized interventions. And importantly, it is predicated on two readily accessible and feasible medical procedures: CXR and routine blood test. It presents a practical pathway toward optimizing the utilization of HRCT, mitigating its associated burden of cost, patient radiation exposure, and the need for specialized infrastructure. This can be a key advantage, particularly in developing regions currently experiencing an increase in engineered stone silicosis cases.

## 5. Conclusions

AI-based techniques that utilize a multimodal fusion approach have been shown to have the potential to outperform single-modality methods and improve automatic staging of silicosis. As a key finding, the integration of two distinct data modalities, structural information from CXRs and biological data from routine blood biomarkers, provided a more complete and accurate diagnostic picture than either modality in isolation. The hybrid and late fusion models achieved the highest and most robust performance, demonstrating a positive performance trend over the strong CXR-based baseline and significantly outperforming the biomarker-only model. The proposed automated multimodal system can serve as an objective decision support tool. It has the potential to reduce the high inter-observer variability that currently challenges the disease staging, ultimately leading to more consistent and timely patient management.

## Figures and Tables

**Table 1:** Pulmonary function metrics during the study, stratified by simple silicosis (SS) and progressive massive fibrosis (PMF). The data are presented as mean values with standard deviations.

Parameter	SS	PMF
FEV_1_ (mL)	3271.77 ± 660.01	2972.25 ± 736.83
FEV_1_ (%)	87.38 ± 13.97	78.35 ± 17.94
FVC (mL)	4256.43 ± 777.93	4099.46 ± 834.28
FVC (%)	90.93 ± 15.54	86.59 ± 15.90
FEV_1_/FVC	0.78 ± 0.05	0.72 ± 0.09
DLCO (mmol/min/kPa)	9.29 ± 2.16	8.79 ± 1.59
DLCO (%)	91.88 ± 21.55	85.48 ± 15.75

Abbreviations: FEV_1_/FVC, Proportion of forced expiratory volume in one second to forced vital capacity; FEV_1_, Forced Expiratory Volume in the first second; FVC, Forced Vital Capacity; DLCO, Diffusing Capacity of Lung for Carbon monoxide.

**Table 2:** Metrics of performance calculated in the ablation study using the image-based unimodal strategy. Results are shown as mean [95% Confidence Interval], segregated by the two lung segmentation approaches. Best AUC per strategy is highlighted in bold.

Segmentation Method	Classifier	Accuracy (%)	F1-Score (%)	AUC
Lung Bounding Box	CatBoost	69.18 [60.96–77.40]	64.59 [58.16–71.02]	**0.77** **[0.68–0.86]**
	RF	69.77 [60.82–78.72]	64.77 [56.40–73.14]	**0.77** **[0.70–0.84]**
	SVM	69.10 [59.02–79.18]	62.84 [52.98– 72.70]	0.74 [0.65–0.83]
Anatomical Rib-Segmentation	CatBoost	75.84 [72.08–79.60]	70.04 [63.65–76.43]	**0.83** **[0.79–0.87]**
	RF	72.01 [65.58–78.44]	65.72 [56.71–74.73]	0.81 [0.77–0.85]
	SVM	74.83 [67.75–81.91]	69.10 [61.59–76.61]	**0.83** **[0.79–0.87]**

Abbreviations: RF, Random Forest; SVM, support vector machines; AUC: area under the receiver operating characteristics (ROC) curve.

**Table 3:** Metrics of performance for the unimodal baselines (image-based and biomarker-based). Results are shown as mean [95% Confidence Interval]. Best AUC per strategy is highlighted in bold.

Strategy	Classifier	Accuracy (%)	Precision (%)	Recall (%)	F1-Score (%)	AUC
Image Based Model	CatBoost	75.84 [72.08–79.60]	80.04 [62.64–97.44]	65.12 [51.97–78.27]	70.04 [63.65–76.43]	0.83 [0.79–0.87]
	RF	72.01 [65.58–78.44]	74.40 [58.08–90.72]	62.48 [45.37–79.59]	65.72 [56.71–74.73]	0.81 [0.77–0.85]
	SVM	74.83 [67.75–81.91]	77.51 [65.42–89.60]	64.45 [49.74–79.16]	69.10 [61.59–76.61]	**0.83** **[0.80–0.88]**
Biomarker Based Model	CatBoost	62.32 [55.06–69.58]	61.81 [40.17–83.45]	57.60 [44.83–70.37]	57.19 [47.77–66.61]	0.69 [0.62–0.76]
	RF	62.66 [55.53–69.79]	61.39 [40.78–82.00]	58.07 [48.55–67.59]	57.76 [48.19–67.33]	**0.70** **[0.64–0.76]**
	SVM	59.75 [52.18–67.32]	60.85 [37.04–84.66]	46.08 [40.84–51.32]	50.46 [43.53–57.39]	0.65 [0.56–0.74]

Abbreviations: RF, Random Forest; SVM, support vector machines; AUC: area under the receiver operating characteristics (ROC) curve.

**Table 4:** Metrics of performance for multimodal approaches. Results are shown as mean [95% Confidence Interval]. Best AUC per strategy is highlighted in bold.

Strategy	Classifier	Accuracy (%)	Precision (%)	Recall (%)	F1-Score (%)	AUC
Early Fusion	CatBoost	75.51 [69.72–81.30]	80.67 [62.54–98.80]	64.14 [50.30–77.98]	69.42 [61.59–77.25]	**0.83** **[0.79–0.87]**
	RF	71.68 [64.51–78.85]	75.58 [58.42–92.74]	62.67 [47.21–78.13]	66.11 [62.26–69.96]	0.82 [0.78–0.86]
	SVM	72.63 [67.61–77.65]	74.62 [61.01–88.23]	63.38 [51.82–74.94]	67.13 [64.41–69.85]	0.82 [0.77–0.87]
Hybrid Fusion	CatBoost	74.37 [69.40–79.34]	79.12 [60.12–98.12]	62.59 [49.53–75.65]	67.96 [60.57–75.35]	**0.85** **[0.80–0.90]**
	RF	71.72 [65.54–77.90]	75.97 [57.19–94.75]	61.55 [48.08–75.02]	65.53 [59.98–71.08]	0.84 [0.80–0.88]
	SVM	73.14 [66.47–79.81]	77.18 [63.82–90.54]	61.66 [46.71–76.61]	66.77 [61.12–72.42]	0.82 [0.77–0.87]
Late Fusion	CatBoost	74.63 [68.96–80.30]	80.34 [62.93–97.75]	62.38 [48.23–76.53]	68.17 [61.85–74.49]	**0.85** **[0.79–0.91]**
	RF	71.80 [64.81–78.79]	76.74 [57.78–95.70]	60.51 [47.04–73.98]	65.34 [58.98–71.70]	0.83 [0.79–0.87]
	SVM	73.62 [66.06–81.18]	79.52 [65.16–93.88]	59.55 [44.49–74.61]	66.37 [58.98–73.76]	0.82 [0.78–0.86]

Abbreviations: SVM, support vector machines; AUC: area under the receiver operating characteristics (ROC) curve.

## Data Availability

The data are not publicly available due to privacy or ethical restrictions. The data that support the findings of this study are available upon request from the corresponding author for researchers who meet the criteria for confidential data access, as stipulated by participant informed consent and the Institutional Research Ethics Committee of the province of Cadiz, Spain. Data requests can be made to this Ethics committee via this email: ceic.hpm.sspa@juntadeandalucia.es.
